# Adenoviral Pharyngitis in the Paediatric Emergency Department: The Pivotal Role of Rapid Antigenic Testing

**DOI:** 10.3390/diagnostics15111306

**Published:** 2025-05-22

**Authors:** Marco Denina, Francesco Del Monte, Emanuele Castagno, Giulia Tosoni, Samuele La Mendola, Federico Vigna, Alessandro Bondi, Angelo Giovanni Delmonaco, Claudia Bondone

**Affiliations:** 1 Paediatric Infectious Diseases Unit, Regina Margherita Children’s Hospital, University of Turin, Città della Salute e della Scienza, 10126 Turin, Italy; mdenina@cittadellasalute.to.it; 2 Department of Pediatric Emergency, Regina Margherita Children’s Hospital, Città della Salute e della Scienza, 10126 Turin, Italy; fdelmonte@cittadellasalute.to.it (F.D.M.); adelmonaco@cittadellasalute.to.it (A.G.D.); cbondone@cittadellasalute.to.it (C.B.); 3 Department of Paediatrics and Public Health, Regina Margherita Children’s Hospital, University of Turin, Città della Salute e della Scienza, 10126 Turin, Italy; giulia.tosoni@unito.it (G.T.); samuele.lamendola@unito.it (S.L.M.); federico.vigna@unito.it (F.V.); 4 Division of Virology, Department of Public Health and Pediatrics, University of Turin, AOU Città della Salute e della Scienza di Torino, 10126 Turin, Italy; abondi@cittadellasalute.to.it

**Keywords:** adenovirus, pharyngitis, rapid antigenic test, paediatric emergency department

## Abstract

**Background:** adenoviruses (AdVs) are DNA viruses that typically cause mild infections in immunocompetent children, and typically involve the respiratory and gastrointestinal tract. Adenoviral pharyngitis is a common paediatric illness, particularly in children under 4 years old. The aim of our 7-year retrospective study, conducted at a tertiary care paediatric emergency department (ED), was to describe the clinical and laboratory characteristics and management of patients with pharyngeal AdV infections. Specifically, we examined how the management of patients with adenoviral pharyngitis has evolved following the introduction of a rapid antigen nasopharyngeal swab test for AdVs, which has been performed directly in the ED since 2023. **Methods:** in this single-centre retrospective observational study, the demographic and clinical information for children discharged from the ED who had been diagnosed with a pharyngeal AdV infection between 1 January 2017 and 31 December 2023 were retrospectively reviewed. Moreover, we compared data before and after the introduction of rapid AdV antigenic swabs, which have been directly performed in the ED since the beginning of 2023. Statistical analysis was undertaken using the Student’s *t*-test and Pearson and Fisher’s exact test, as appropriate. Significance was set at *p*-value < 0.05. **Results**: during the study period, 172 children were diagnosed with adenoviral pharyngitis based on a positive swab. All patients were febrile, with a median duration of fever of 4 days. Blood tests were requested for 84.9% of patients at admission, resulting in a mean WBC count of 13,250/mmc and a mean CRP of 70.6 mg/L. The highest CRP median values were found on the third day of fever. Out of 383 swabs performed during 2017–2022, 13.6% were positive vs a 32% positive rate for the 372 swabs performed in 2023. The mean duration of observation in the ED before 2023 was 31.4 h vs. 10.4 h in 2023. Similarly, 9% of patients with adenoviral pharyngitis were admitted to a paediatric ward before 2023 and only 0.8% in 2023. **Conclusions:** the primary reason for ED admission in cases of adenoviral pharyngitis is fever lasting several days due to hyperinflammation. Differential diagnosis with bacterial infection is essential to limit the number of hospitalisations and inappropriate antibiotic therapy. The introduction of the rapid antigen nasopharyngeal swab has simplified the diagnosis of adenoviral pharyngitis, enabling timely and accurate differentiation from bacterial causes.

## 1. Introduction

Adenoviruses (AdVs) generally cause mild respiratory, gastrointestinal (GI), and conjunctival infections in immunocompetent children [1].

Currently, 104 different AdV serotypes are known, and these have been classified into seven species: A to G. Most AdV types belong to species D (73 types), followed by species B (16 types), but new AdV types continue to emerge. Different serotypes show different tissue-tropism, which is related to different clinical features [2,3].

Human AdV infections are more common in childhood. Over 80% of diagnosed AdV infections occur in children under 4 years due both to their lack of humoral immunity and to community life with other children in closed environments such as nurseries or kindergartens [1,2,3].

The incidence and prevalence of AdV infections are unknown because of their self-limiting nature, and often patients do not visit a doctor and do not undergo any microbiological test [4]. Most epidemics occur in winter or early spring, but infections may occur throughout the year, with no apparent seasonality [1,2,3,5].

Asymptomatic carriage of AdV may persist for weeks or months [1,3]. Transmission of AdVs can occur via droplet inhalation, direct conjunctival inoculation, faecal–oral spread, exposure to infected tissue or blood, or environmental surfaces. The incubation period ranges from 2 to 14 days and depends upon viral serotype and the mechanism of transmission [1,3].

It has been estimated that AdVs accounts for at least 5 to 10% of paediatric respiratory tract infections (RTIs) [3]. Typical features include fever, pharyngitis, tonsillitis, cough, and sore throat; GI symptoms may be present concomitantly. In immunocompetent patients, symptoms usually abate spontaneously within 2 weeks, and infection induces type-specific immunity [3].

Pharyngitis is a common reason for paediatric examination. While Streptococcus is the most significant bacterial cause of acute pharyngitis, viruses are the predominant cause, including Influenza, Parainfluenza, Coronaviruses, Enteroviruses, Rhinoviruses, Respiratory Syncytial Virus, Cytomegalovirus, Epstein–Barr virus (EBV), Herpes Simplex Virus, and Human Metapneumovirus, along with AdVs [6].

Traditionally, the diagnosis of adenoviral infection relied on viral culture, which, although sensitive, typically requires several days to yield results and is, therefore, less suitable for urgent clinical decision-making. In current practice, a real-time polymerase chain reaction (RT-PCR) has largely replaced viral culture due to its higher sensitivity and shorter turnaround time. However, RT-PCR still requires laboratory infrastructure and several hours to produce results. Therefore, rapid antigen tests based on fluorescence immunoassay technology represent a valuable alternative for the detection of adenoviral infections within minutes—particularly in paediatric emergency departments, where timely clinical decisions are essential [7].

Although most cases of adenoviral pharyngitis are self-limited, many children come to the ED and receive unnecessary antibiotic therapy [5]. Experimental studies have shown no effective treatment for this infection, and only supportive care is indicated [8,9].

Our seven-year retrospective study, conducted at a tertiary care paediatric ED, aimed to describe the clinical and epidemiological characteristics, admission features, management of, and outcome for, patients with a pharyngeal AdV infection. Our work also aimed to evaluate the impact of a rapid bedside adenoviral antigenic swab on patient flow in the paediatric ED.

## 2. Materials and Methods

This single-centre retrospective observational study was conducted at the paediatric ED of the Regina Margherita Children’s Hospital in Turin, Italy. Demographic and clinical information were retrospectively extracted from electronic health records of children discharged from the ED who had been diagnosed with pharyngeal AdV infection between 1 January 2017 and 31 December 2023. The following diagnosis codes (International Classification of Diseases, 9th revision, ICD-9) were considered: “462—Acute pharyngitis”, “463—Acute tonsillitis”, and “079.0 —Adenovirus infection in conditions classified elsewhere and of unspecified site”. We also searched for the following procedures: “LLU18032-TF Adenovirus antigen pharyngeal swab” and “LLU16046-TF Adenovirus DNA swab”.

The medical record databases in our hospital were searched for information on the children’s demographic profile (age and sex), duration of fever on admission, white blood cell (WBC) and C-reactive protein (CRP) levels, and outcome (discharge from the ED, monitoring at the paediatric Short Observation Unit (SOU), hospitalisation, and readmission). We also recorded data on therapeutic management, such as corticosteroid administration, antibiotic treatment (before and after the swab results), or no treatment.

The diagnosis of AdV infection was based on a positive AdV nasopharyngeal swab. Until the end of 2022, all the swabs were processed in the hospital laboratory using either a molecular or antigenic test: Adeno Respi Card (Mascia Brunelli S.p.a, Milan, Italy), a rapid immunochromatographic test, was used for the qualitative determination of Adenovirus antigens, while the QIAsymphony SP instrument (QIAGEN, Hilden, Germany) paired with the ABI 7500 Fast Dx Real-Time PCR Instrument (ELITechGroup, Turin, Italy) were used to detect the Adenovirus DNA from nasopharyngeal swabs. The molecular test results were available after several hours, or sometimes the day after. Since 2023, the laboratory swab was substituted by a bedside antigenic test performed directly in the ED: the STANDARD F2400 analyser (SD BIOSENSOR, Suwon, Republic of Korea), based on fluorescence-based lateral flow immunoassay technology, was used paired with the Adeno Respi Ag FIA test produced by the same company, for the detection within 15 min of AdV antigens in nasopharyngeal swabs. Based on the manufacturer’s clinical evaluation, the sensitivity of the test is 83.3% and the specificity is 95.5%. Additionally, the positive predictive value (PPV) is approximately 94.9%, and the negative predictive value (NPV) is 85%. Recently, Romero-Gomez MP, et al. reported a sensitivity of 71.93% and specificity of 100% [10].

All clinical information was collected with full respect being given to the children’s and their families’ privacy. In accordance with European regulations, Italian observational studies from data obtained without any additional therapy or monitoring procedures do not need the approval of an Independent Ethical Committee.

The statistical analysis was performed using IBM SPSS Statistics 26.0 (IBM Corp., Armonk, NY, USA). In the first part of the study, we conducted a descriptive analysis; categorical variables are reported as absolute numbers and percentages, and continuous variables as median and interquartile range (IQR), as appropriate. Student’s *t*-test was used to compare the continuous variables of the study groups. Pearson and correlation Fisher exact tests were performed to evaluate the discrete variables, as appropriate. Significance was set at *p*-value < 0.05.

## 3. Results

During the study period, 4045 cases of acute pharyngotonsillitis were recorded. An AdV was suspected in 755 patients (18.7%), and an AdV nasopharyngeal swab was performed. In 172 cases, a positive swab allowed the diagnosis of adenoviral pharyngitis (22.8% of total AdV antigen swabs and 4.3% of overall pharyngotonsillitis).

**Figure 1:**
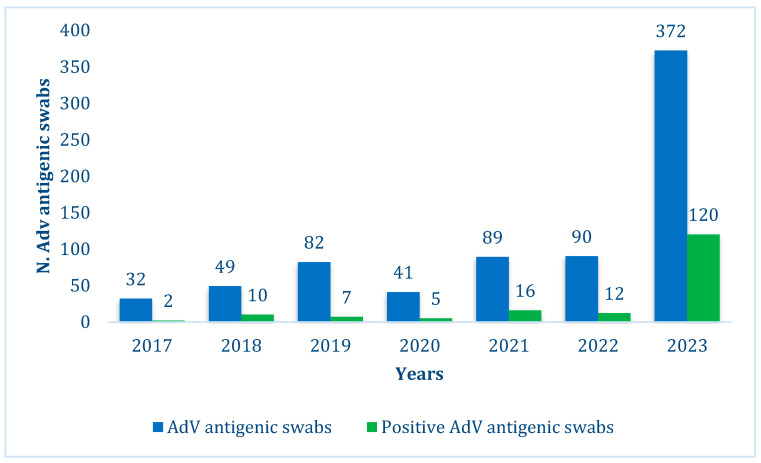
Number of total AdV swabs performed (blue) and positive swabs (green) over the years.

Out of the 383 swabs performed between 2017 and 2022, 52 (13.6%) were positive vs. 120/372 bedside swabs (32%) performed in 2023 (Figure 1).

**Figure 2:**
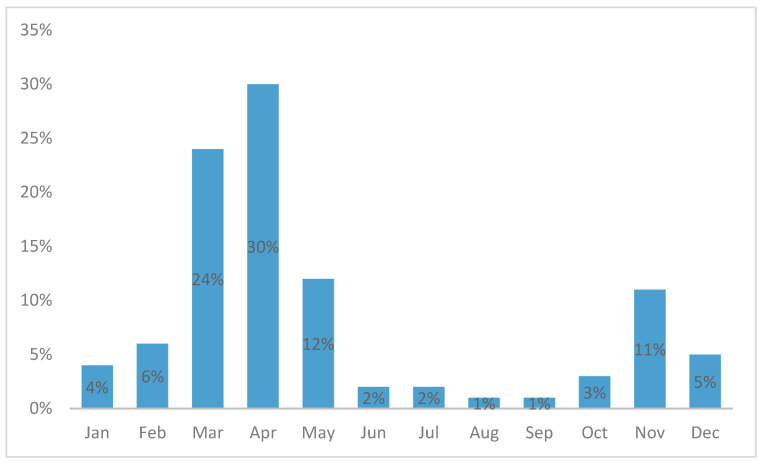
Seasonal pattern of all pharyngeal AdV cases.

The incidence was higher in the winter–spring season, with a peak in April (Figure 2).

Males accounted for 58.7% of cases, and the median age was 2.9 years (IQR 1.5–5.1 years).

All patients were febrile, the median duration of fever was 4 days (IQR 2–5 days). Antibiotic therapy had already been started in 84 patients (48.8%). Ninety per cent of cases of AdV pharyngitis were admitted with a green low-priority code.

Blood tests were performed on most patients at admission (84.9%), resulting in a median WBC count of 13,250/mm^3^ (IQR 10,075/mm^3^–17,700/mm^3^) and a median CRP level of 70.6 mg/L (IQR 46.55–104.4 mg/L).

Clinical and laboratory data for the 172 study patients are listed in Table 1.

Twenty-six (15.1%) patients were also tested for group A beta-haemolytic Streptococcus (GABHS) infection with a rapid antigenic pharyngeal swab, and 32 (18.6%) patients were also tested for EBV infection with a serological test. None proved positive.

A single dose of oral betamethasone was administered in 158 cases (91.9%) of AdV pharyngitis. Among them, 24 patients (13.9%) were discharged with ongoing antibiotic therapy, 53 patients (30.9%) discontinued ongoing antibiotic therapy, 79 patients (45.9%) received no antibiotic therapy either before or after discharge, and two patients (1.2%) received a new antibiotic prescription.

Fourteen patients (8.1%) with Adv pharyngitis were not treated with betamethasone. Among them, six patients (3.5%) were discharged with ongoing antibiotic therapy, while eight patients (4.6%) were discharged without any treatment: in this last group, two patients (1.1%) discontinued ongoing antibiotic therapy while six patients (3.5%) received no treatment, either before or after discharge.

For 55 patients, representing 65% of all those on antibiotic therapy, antibiotics were discontinued after a positive swab. Antibiotic therapy was continued for 29 patients (35% of those on antibiotic therapy) and was added for 2 patients after the swab result. Six patients (3.5% of all AdV pharyngitis) were discharged without treatment.

Ninety-four patients (54.6%) were discharged home directly from the ED. Only six children were hospitalised (3.5%). However, 72 patients (41.9%) continued to be monitored at the paediatric SOU for less than 36 h. In 14 cases (8.1%), the patient returned to the ED within 72 h for the same problem. Table 2 reports data on the management of patients.

**Figure 3:**
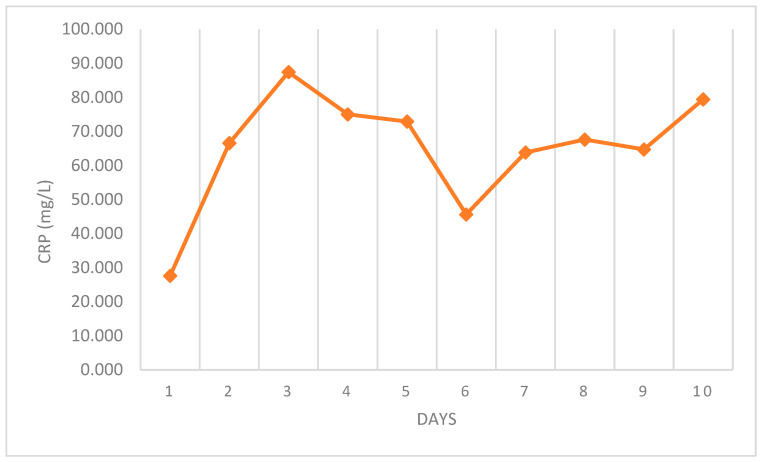
Relationship between the duration of fever and the mean CRP values.

The evaluation of the relationship between CRP value and the duration of fever showed that the highest median values were found on the third day of fever; however, this was not statistically significant (*p* = 0.314) (see Figure 3).

The mean CRP level was similar in patients who were already on antibiotic therapy at admission (89.69 mg/L) and in those who were not (62.32 mg/L) (*p* = 0.375), and was similar in those discharged with antibiotic therapy (82.25 mg/L) compared to those discharged without (75.28 mg/L) (*p* = 0.806). The CRP level in hospitalised patients was not significantly lower (mean CRP 57.88 mg/L) than in discharged patients (mean CRP 77.56 mg/L) (*p* = 0.232).

Patients admitted to the ED with ongoing antibiotic therapy had a longer duration of fever (4.4 days) compared to those without antibiotics (3.6 days), *p*-value of 0.931. Slightly younger patients (mean age 2.5 years) were admitted to paediatric wards when compared to those who were discharged (mean 3.7 years), with a *p*-value of 0.718. In the same way, slightly younger patients returned more frequently to the ED compared to those who did not return (mean age 3.1 years vs. 3.6 years) (*p* = 0.663).

After introducing rapid bedside swabs in 2023, we observed a shorter duration of observation in the ED, lower admission rate to the SOU, lower hospitalisation rates, and fewer readmissions. Of the 72 patients who needed observation in the SOU, 65% were admitted before 2023 (*p* < 0.001). On the other hand, we observed a higher positivity rate of swabs and a higher prescription level of betamethasone (Table 3). Blood tests were performed on all patients upon admission before 2023 and on 78% of patients in 2023.

## 4. Discussion

In this retrospective study, we analysed the characteristics of patients admitted to the paediatric ED of a tertiary care Children’s Hospital who were diagnosed with adenoviral pharyngitis.

Our population showed a slight male-to-female prevalence consistent with previous studies regarding AdV infections [5,7,11]. The median age of patients in our study was 40 months, and most patients were between 1 and 5 years old, which aligns with the literature [5,11]. The seasonal distribution confirmed previous studies, with a peak in spring and high incidence in the winter months, but with infections occurring all year round [1,2,5,11].

Fever was the main symptom of adenoviral pharyngitis and the main reason for admission to the ED [1,5,11,12]. The median duration of fever at admission was 4 days, which is consistent with the literature [3,12]. Overall, 84% of patients who entered the ED had already begun antibiotic treatment. This may be because distinguishing between AdVs and bacterial infections can be challenging without specific diagnostic tests [5]. Patients with fever for several days were more likely to receive antibiotic therapy, although the results did not reach statistical significance. Antibiotics were stopped after a positive swab in 65% of patients already put on antibiotics: we can argue that early diagnosis of AdV infection could have allowed doctors to avoid inappropriate antibiotic therapy.

Blood tests are largely used in the ED since they can help distinguish between viral and bacterial infection, and CRP performs better in predicting severe bacterial infection in young children than WBC count [12]. In our study, 84.9% of patients underwent a blood test, and the medial value of CRP was 70.6 mg/L (IQR 46.55–104.4 mg/L), which is consistent with the literature. However, if clinical decision-making strongly relied on CRP values, many AdV infections would be misclassified as bacterial infections and managed accordingly. In fact, although elevated CRP generally reflects bacterial infection, Appenzeller et al. reported that AdVs show a clearly high mean CRP level (49 mg/L), higher than other viral respiratory tract infections such as the Influenza virus (23 mg/L) [12]. Putto et al. found that mean CRP levels in tonsillitis caused by AdVs, EBV, and GABHS were similar [13]. EBV and GABHS were also searched for in our population along with AdVs, given the similar clinical presentation of pharyngotonsillitis with elevated CRP levels in 18.6% and 15.1% of patients, respectively.

The inflammatory events leading to increased CRP concentrations in adenoviral infections are still not completely understood [5,14,15,16]. An explanation could be bacterial co-infection; however, several scientific studies have refused this hypothesis [14,15]. In our case, median CRP levels were higher in patients already put on antibiotic therapy. Although there were no statistically significant differences, this suggests that CRP could not be due to bacterial co-infection, as otherwise, it would have been reduced during antibiotic therapy. On the contrary, an increased CRP value might be related to the high interleukin-6 (IL-6) values found in patients with adenoviral respiratory infections [14,15,16]. AdVs induce a prominent acute-phase response, including recruiting neutrophils, lymphocytes, macrophages, and natural killer cells to the site of infection and elaborating various cytokines and chemokines [3]. Kawasaki et al. found a correlation between the serum concentrations of CRP and IL-6. Serum IL-6 concentrations in patients with adenoviral respiratory infection were higher than those in patients with other viral respiratory tract infections (like influenza virus and respiratory syncytial virus infection) [16]. Sun et al. reported that elevated IL-6 levels were associated with severe or fatal adenoviral infections, whereas there was no association between CRP levels and severity of infection [14]. Consistent with this, even if not statistically significant, in our study, the mean CRP was higher in those discharged home and lower in those hospitalised, confirming that a correlation between CRP levels and severity of illness is unlikely. Moreover, we found a higher level of CRP (although not statistically significant) on the third day of fever (Figure 2). This may reflect the peak of the inflammatory response.

At the beginning of 2023, rapid bedside AdV antigenic swabs directly performed in our paediatric ED were introduced. Comparing the results obtained before and after 2023, we demonstrated an increased incidence of AdV infection in 2023. This may be due to increased circulation of the virus and susceptible patients, which is related to the poor circulation of the virus in the years of the COVID-19 pandemic and in the same period for other viruses [17]. However, the number of swabs performed in 2023 increased due to the introduction of rapid bedside swabs, allowing diagnosis to be made within a few minutes. In the years 2017–2022, 383 swabs were taken, and 52 were positive (13.6%). In 2023, 372 rapid swabs were taken, and 120 were positive (32%). The difference was statistically significant. One possible explanation is that during 2017–2022, many cases of AdV pharyngitis were probably managed based on clinical suspicion alone, without performing swab tests for confirmation. Before 2023, AdV swabbing was mainly used to rule out AdV infection rather than to confirm it.

Early detection of AdVs has also led to a significant reduction in observation time in the ED, as well as the need for admission to the SOU and paediatric units [18,19]. The reason for hospitalisation was the longer time it took to receive the swab result. Out of the 47 patients, 31 were diagnosed with an AdV during their observation in the SOU. The number of return visits to the ED has also been significantly reduced. Early diagnosis also reduced the need for blood tests.

Based on experimental studies, currently there are no effective treatments for adenoviral infection in immunocompetent patients. The administration of betamethasone in a single dose is reported to be effective to reduce significantly the hyper-inflammation that the virus can cause in children [9]. However, although its prescription is encouraged by positive clinical experience, the use of this therapeutic practice varies due to lack of experimental evidence. A retrospective observational study on 23 children hospitalised for tonsillitis concluded that a single dose of betamethasone 0.1 mg/kg resulted in almost immediate relief within 12 h [9]. This observation confirms that maybe an AdV itself is not the cause of prolonged febrile syndrome. Instead, symptoms might be due to the hyperinflammatory response triggered by the immune system. A single dose of betamethasone could reduce this hyperinflammatory response, but evidence is still poor.

In our study, almost all patients with an AdV infection were treated with a single dose of betamethasone 0.1 mg/kg. Betamethasone was administered to 97.5% of patients in 2023 and to 79% of patients during 2017–2022 (*p*-value < 0.05). Although no strong evidence supports its use, our group has increasingly used betamethasone based on clinical experience and practice in recent years. It is worth noting that betamethasone is considered a safe drug, and its use in single doses is not associated with an increase in side effects [20]. AdV infection is typically self-limiting and unlikely to cause severe symptoms in immunocompetent patients. However, effective treatment can lead to excellent public health outcomes, such as reducing the duration of illness and hospitalisation.

Our study has some strengths. First, we evaluated numerous cases of AdV pharyngitis, a topic with limited coverage in the literature. Moreover, we first analysed the benefits of the rapid AdV bedside antigen swab on the clinical management of children admitted to the ED.

The study also has some limitations: first, it was single-centre and retrospective. It is possible that some data may have been lost, as not reported in hospital records: we included only patients with a positive swab for AdVs, and before 2023, some patients might have been managed on a clinical basis without undergoing a swab test, losing their data. Furthermore, direct biomarkers (e.g., IL-6) or other evidence excluding bacterial co-infections has not been investigated, our article reflects the real-word experience of a paediatric emergency department, in which IL-6 dosage is inaccessible, haemoculture is not routinely asked for, excepting when suspecting invasive bacterial infections, and only WBC, CRP, and PCT are available to help clinicians distinguish between viral and bacterial infections. Furthermore, our data did not include AdV swabs from inpatients.

Last, in our real-world setting, the rapid antigen test was not routinely duplicated with molecular PCR testing, in accordance with institutional stewardship policies aimed at ensuring appropriate test utilization and avoiding unnecessary resource consumption. Furthermore, viral culture was not considered a viable option in our ED, due to its prolonged turnaround time and the high volume of children visited daily. Given these constraints, we did not generate a dataset suitable for traditional diagnostic accuracy analysis. Future prospective studies specifically designed to assess the test’s sensitivity and specificity in paediatric emergency settings would certainly be valuable and are warranted.

## 5. Conclusions

AdV pharyngitis is a common paediatric illness, particularly in children under 4 years of age. Our 7-year retrospective study confirms epidemiological and laboratory data consistent with the previous literature. The primary reason for ED admission was fever lasting several days due to hyperinflammation. The introduction of rapid bedside adenoviral antigenic testing has proven to be a valuable tool in everyday paediatric emergency practice. As clearly demonstrated by our results, the use of rapid antigenic swabs has enabled a more timely and accurate diagnosis, allowing clinicians to reduce unnecessary blood tests, inappropriate antibiotic prescriptions, length of ED observation, hospital admissions, and return visits. These findings support the broader implementation of point-of-care testing for Adenovirus to improve clinical management, reduce healthcare costs, and promote antibiotic stewardship.

## Figures and Tables

**Table 1:** Demographic, anamnestic, and laboratory characteristics of the study population.

	N. Adenoviral Pharyngitis (Total 172)
Sex (%)	
Male	101 (58.7)
Female	71 (41.3)
Age (years)	2.9 * [1.5–5.1] **
Findings at presentation	
Duration of fever (days)	4 * [2–5] **
Antibiotic therapy (%)	84 (48.8)
CRP (mg/L)	70.6 * [46.55–104.4] **
WBC/mm^3^	13,250 * [10,075–17,700] **

* Median values, ** IQR interquartile range. WBC: white blood cell count, CRP: C-reactive protein.

**Table 2:** Management of patients.

	N. Adenoviral Pharyngitis (%)
Triage priority code	
Green (low priority)	156 (91%)
Yellow (high priority)	14 (8%)
Red (emergency)	2 (1%)
Laboratory test	
Blood test	146 (84.9%)
GABHS test	26 (15.1%)
EBV test	32 (18.6%)
Therapy	
Betamethasone	158 (91.9%)
No antibiotic	79 (45.9%)
Antibiotic continuation	24 (13.9%)
Antibiotic discontinuation	53 (30.9%)
New antibiotic prescription	2 (1.2%)
No betamethasone	14 (8.1%)
Antibiotic continuation	6 (3.5%)
Antibiotic discontinuation	2 (1.1%)
No therapy	6 (3.5%)
Outcome	
Discharged	94 (54.6%)
SOU	72 (41.9%)
Hospitalisation	6 (3.5%)
Readmission	14 (8.1%)

GABHS: group A beta-haemolytic Streptococcus; EBV: Epstein–Barr virus; SOU: Short Observation Unit.

**Table 3:** Statistically significant differences between the years 2017–2022 and 2023.

	2017–2022	2023	*p*-Value
N. AdV antigenic swabs	383	372	
Positive swabs (%)	52 (13.6%)	120 (32%)	<0.001
Duration of observation (mean hours)	31.4	10.4	0.015
N. admission to SOU (%)	47 (90%)	25 (21%)	<0.001
N. hospitalisation (%)	5 (9.6%)	1 (0.8%)	0.004
N. readmission (%)	9 (17%)	5 (4%)	0.013
N.betamethasone prescriptions (%)	41 (79%)	117 (97.5%)	<0.001

## Data Availability

The data presented in this study are available on request from the corresponding author.

## Abbreviations

The following abbreviations are used in this manuscript:

AdVs	Adenoviruses
ED	Emergency department
WBC	White blood cell
CRP	C-reactive protein
SOU	Short Observation Unit
GI	gastrointestinal
RTIs	respiratory tract infections
IQR	interquartile range
GABHS	group A beta-haemolytic streptococcus
EBV	Epstein–Barr virus
